# Orbital Plasmacytoma in an Elderly Patient With Multiple Myeloma

**DOI:** 10.7759/cureus.53823

**Published:** 2024-02-08

**Authors:** Salma Aloui, Pir Abdul Ahad Aziz Qureshi, Vikram Rao Bollineni

**Affiliations:** 1 Department of Radiology, Universitair Ziekenhuis Brussel, Brussels, BEL; 2 Department of Radiology, Landspítali - The National University Hospital of Iceland, Reykjavík, ISL

**Keywords:** secondary malignancies, mri, ct, radiological features, multiple myeloma, orbital plasmacytoma

## Abstract

Orbital plasmacytomas are exceedingly rare neoplasms characterized by the proliferation of monoclonal plasma cells in the orbital soft tissues. Their presentation and clinical course can be diverse, making early diagnosis and management challenging. This case report sheds light on one such instance, emphasizing the diagnostic and therapeutic aspects of this uncommon condition. We present an 81-year-old patient with a prior diagnosis of multiple myeloma and bilateral orbital plasmacytomas, highlighting the importance of imaging in diagnosis and management.

## Introduction

Plasmacytoma, a pathological proliferation of plasma cells, represents a subtype of plasma cell dyscrasia, which can present itself as multiple myeloma, primary amyloidosis, or monoclonal gammopathy of undetermined significance (MGUS). Plasmacytoma can be primary or secondary to disseminated multiple myeloma, categorizing it into two distinct groups, i.e., medullary (about 2-5% of all plasma cell neoplasms) and extramedullary (about 3% of all plasma cell neoplasms). Extramedullary plasmacytoma usually manifests in the upper respiratory, head, and neck regions [1].

## Case presentation

**Figure 1:**
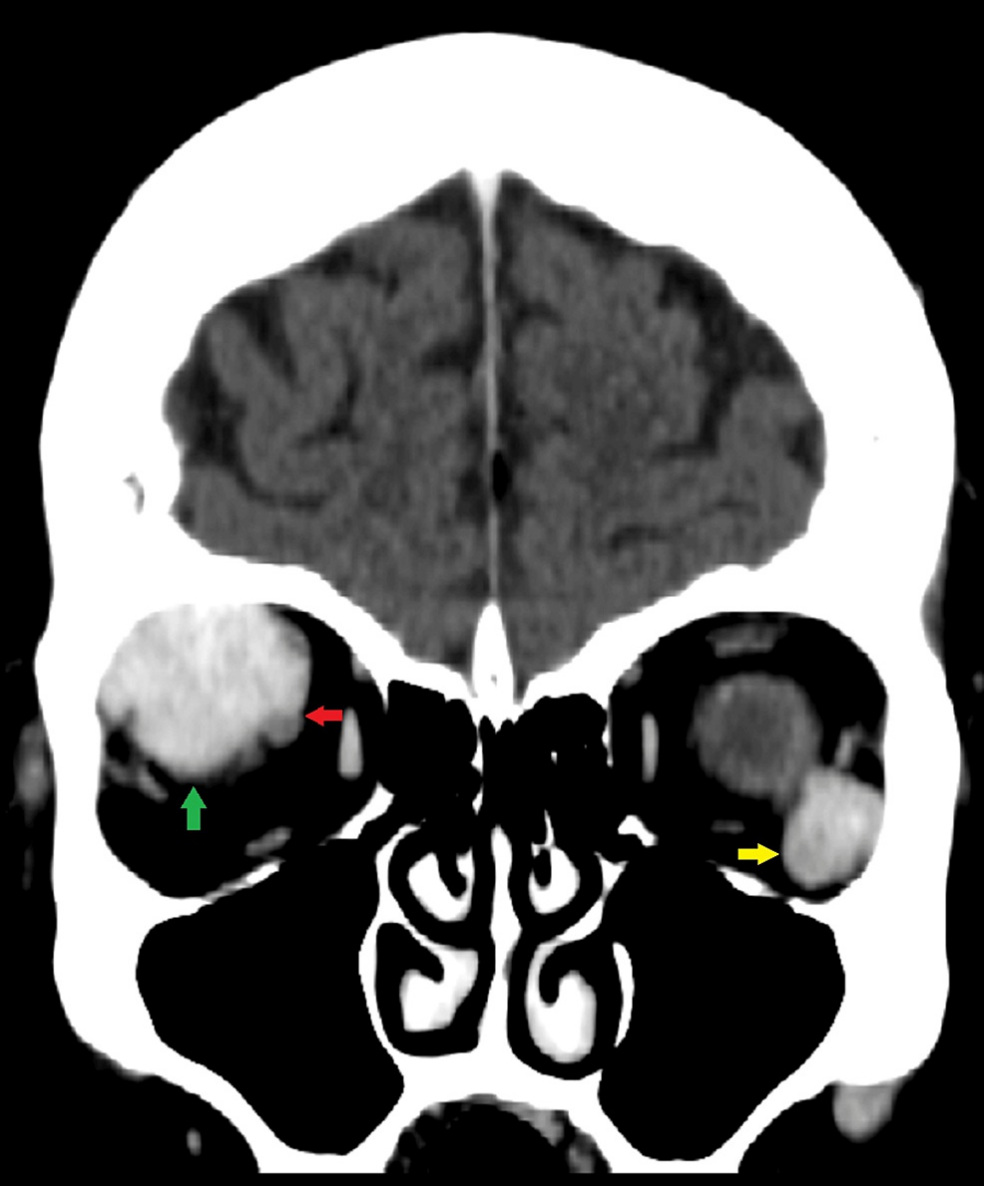
CT brain scan with contrast (coronal view) Well-defined, bilateral intra-orbital tumors with homogenous contrast enhancement are seen (green and yellow arrows). The lesion in the right orbit is encasing and compressing the right optic nerve (red arrow).

An 81-year-old female patient who was diagnosed with multiple myeloma in 2019 presented with a three-month history of double vision, bulging of the right eye globe, restricted eye movement, and headache. On general physical examination, she had a high blood pressure of 160/95 mm Hg, and her pulse rate was 80 b/min. An ophthalmological examination revealed bilateral diplopia in all quadrants except inferiorly, mild proptosis of the right eye, and pupils that were normal bilaterally. Subsequently, a CT scan of the brain was advised to evaluate the patient´s symptoms, which revealed well-defined bilateral intra-orbital tumors with homogenous contrast enhancement. No overlying bone erosion or destruction was identified. The largest mass was in the right retrobulbar region, measuring 26 mm (Figure 1).

**Figure 2:**
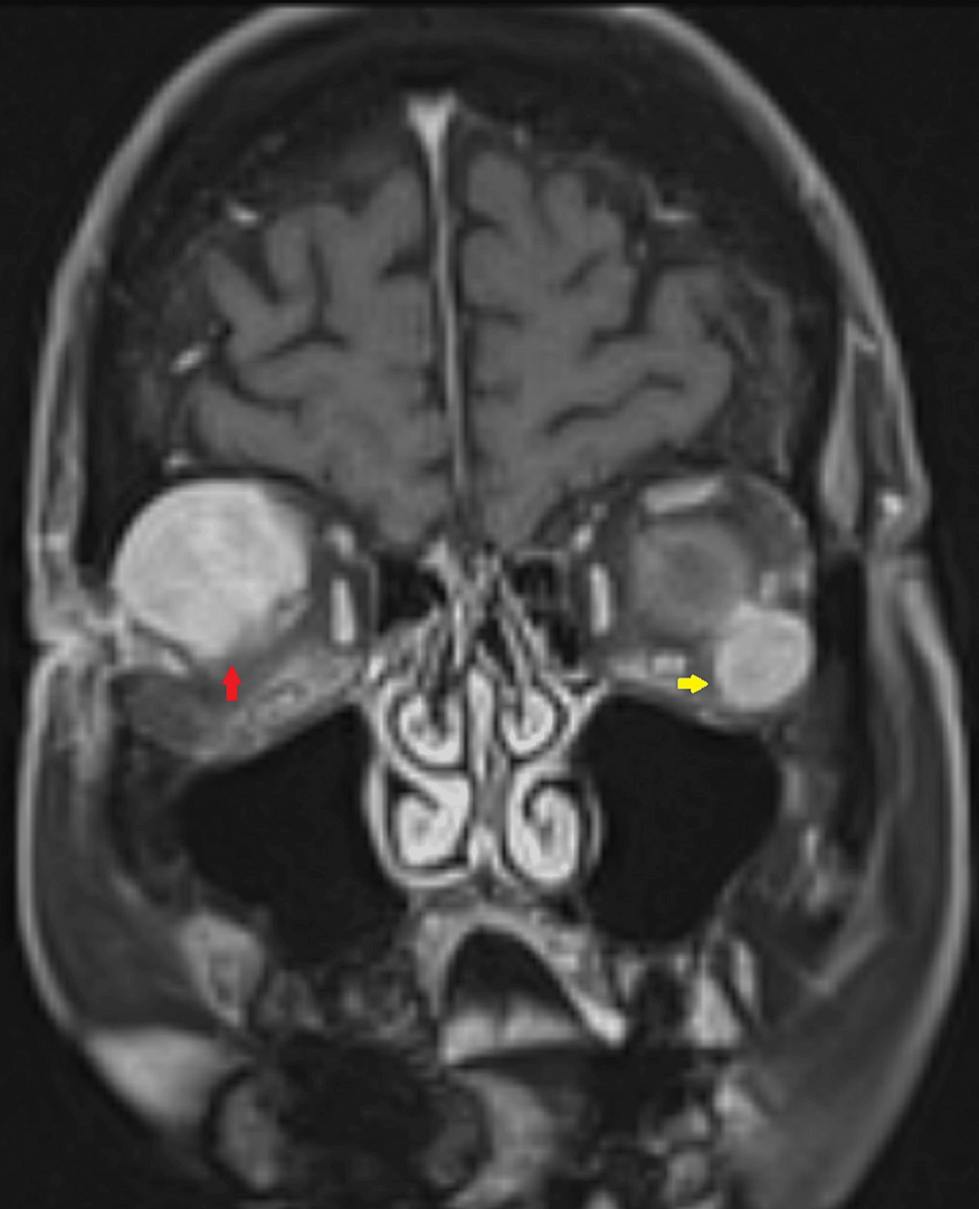
T1 post-contrast brain MRI image (coronal view) Uniformly enhancing, bilateral intra-orbital lesions are seen (red and yellow arrows). The lesions are closely abutting the adjacent extraocular muscles. Note the larger lesion in the right orbit encasing the optic nerve and compressing and displacing it inferiorly.

Additionally, there were multiple scattered osteolytic lesions in the skull, spine, sternum, and probably in the ribs, pelvis, and scapula on the CT scan. An MRI was performed to better evaluate the lesions, their extent, and their relationship with surrounding structures. It identified three orbital well-circumscribed, homogeneously enhancing, diffusion-restrictive soft tissue lesions, with the largest lesion in the right eye measuring 4 cm, encasing the optic nerve with focal loss of intervening fat planes and exerting a mass effect on the conus (Figure 2).

Based on the clinical history and imaging findings, the differentials of orbital plasmacytoma, lymphoma, and metastases were advised. Subsequently, the patient underwent excision of the lesions, and histopathology of the specimen revealed plasma cell infiltration in soft tissue where the cells are partly atypical and many binucleated plasma cells are seen. Appearance was compatible with plasmacytoma.

The patient was then further treated with chemotherapy and radiotherapy. Currently, the patient is being treated with chemotherapy and receiving bortezomib once a month and lenalidomide 10 mg for seven days every other week, which has kept the disease in remission clinically. No further imaging was requested by the treating physician at this point, and the patient is advised for monthly follow-up.

## Discussion

Radiological assessment of orbital plasmacytomas often involves CT and MRI scans. Orbital plasmacytomas are typically localized within the orbital soft tissues, and imaging can visualize the tumor's location within the orbit. On CT and MRI scans, orbital plasmacytomas often appear as well-defined, enhancing soft tissue masses within the orbital space. These masses may be homogeneous in appearance.

When administering contrast, orbital plasmacytomas typically exhibit enhancement, signifying increased vascularity within the lesion. One distinctive characteristic of orbital plasmacytomas is their capacity to induce bone destruction. Imaging reveals this as erosive changes in the adjacent orbital bones, representing an essential distinguishing factor from other orbital tumors [2-4].

In some cases, these tumors may infiltrate the surrounding orbital soft tissues, leading to displacement or compression of the adjacent structures, which is evident in imaging. Imaging plays a crucial role in monitoring the response to treatment. Following treatment (e.g., surgery and/or radiotherapy), serial imaging may show a reduction in the lesion size and a decrease in enhancement, indicating a positive response to the treatment [5].

In clinical practice, radiological assessment of orbital plasmacytomas plays a valuable role in initial evaluation but has inherent limitations. These limitations include the non-definitive nature of imaging findings, their potential lack of specificity that can lead to misdiagnosis, and the challenge of distinguishing between tumor recurrence and post-treatment effects over time. Furthermore, it is crucial to consider a range of differential diagnoses, including metastasis, vascular malformations, lymphoma, inflammatory conditions, and benign lesions. These conditions may share radiological characteristics with plasmacytomas, emphasizing the need for a multidisciplinary approach and histopathological confirmation to ensure precise diagnosis and appropriate management.

## Conclusions

The coexistence of orbital plasmacytomas with multiple myeloma poses a diagnostic challenge and demands a multidisciplinary approach. Characterizing the extent of the orbital lesion and guiding treatment strategies through radiological assessment becomes crucial. In this case, focal radiation therapy effectively treated the patient, resulting in complete remission. The radiological characteristics of orbital plasmacytomas can vary from case to case. However, the presence of a well-defined, enhancing mass with bone destruction and soft tissue infiltration often serves as an indicative feature of this rare orbital neoplasm. Radiological evaluation is critical for initial diagnosis, surgical planning, and post-treatment monitoring of orbital plasmacytomas.
